# Beneficial Effects of Rosmarinic Acid on IPEC-J2 Cells Exposed to the Combination of Deoxynivalenol and T-2 Toxin

**DOI:** 10.1155/2020/8880651

**Published:** 2020-12-22

**Authors:** Judit Mercédesz Pomothy, Réka Fanni Barna, Erzsébet Anna Pászti, Ákos Babiczky, Áron Szóládi, Ákos Jerzsele, Erzsébet Pásztiné Gere

**Affiliations:** ^1^Department of Pharmacology and Toxicology, University of Veterinary Medicine Budapest, H-1078 Budapest, Hungary; ^2^Department of Physiology and Biochemistry, University of Veterinary Medicine Budapest, H-1078 Budapest, Hungary; ^3^Neuronal Networks and Behaviour Research Group, Research Centre for Natural Sciences, H-1117 Budapest, Hungary; ^4^Faculty of Natural Science, Budapest University of Technology and Economics, H-1111 Budapest, Hungary

## Abstract

Mycotoxin contamination in feedstuffs is a worldwide problem that causes serious health issues both in humans and animals, and it contributes to serious economic losses. Deoxynivalenol (DON) and T-2 toxin (T-2) are major trichothecene mycotoxins and are known to challenge mainly intestinal barrier functions. Polyphenolic rosmarinic acid (RA) appeared to have antioxidant and anti-inflammatory properties *in vitro*. The aim of this study was to investigate protective effects of RA against DON and T-2 or combined mycotoxin-induced intestinal damage in nontumorigenic porcine cell line, IPEC-J2. It was ascertained that simultaneous treatment of DON and T-2 (DT2: 1 *μ*mol/L DON + 5 nmol/L T − 2) for 48 h and 72 h reduced transepithelial electrical resistance of cell monolayer, which was restored by 50 *μ*mol/L RA application. It was also found that DT2 for 48 h and 72 h could induce oxidative stress and elevate interleukin-6 (IL-6) and interleukin-8 (IL-8) levels significantly, which were alleviated by the administration of RA. DT2 administration contributed to the redistribution of claudin-1; however, occludin membranous localization was not altered by combined mycotoxin treatment. In conclusion, beneficial effect of RA was exerted on DT2-deteriorated cell monolayer integrity and on the perturbated redox status of IPEC-J2 cells.

## 1. Introduction

**Figure 1:**
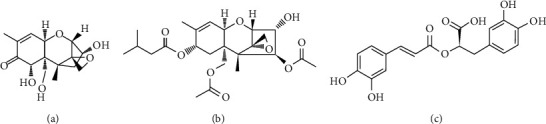
Chemical structures of the tested food-borne compounds: (a) DON, (b) T-2 toxin, and (c) RA.

Mycotoxins are secondary metabolites produced by various fungi. Human and farm animal exposure to *Fusarium* mycotoxins such as deoxynivalenol (DON) and T-2 toxin (T-2) could occur via feedstuff ingestion (Figures 1(a) and 1(b)).

The enterocytes serve as a pivotal barrier between the organism and numerous noxious stimuli. These cells absorb the nutrients and form a border against the pathogens and toxins and actively take part in the modulation of the immune functions of the gut [1]. The IPEC-J2 cell line model is a noncancerous, nontransformed cell model suitable for *in vitro* studying on interaction between xenobiotics such as lipopolysaccharide [2, 3] or mycotoxin [4, 5] and intestinal epithelium.

Several research groups reported that DON application results in significant reduction in transepithelial electrical resistance (TEER) values in a dose- and application route-dependent manner [6–8]. T-2 could also decrease IPEC-J2 cell barrier integrity at 210 nmol/L for 72 h [7].

Adjacent enterocytes close their paracellular space around themselves with forming tight junctions (TJ). It was also investigated if there is a connection between mycotoxin contamination and expression of TJ proteins of intestinal cell monolayers. Zonula occludens- (ZO-) 1 expression was impacted differently when epithelial cells of porcine small intestinal origin were exposed to low or high concentrations of DON. Disintegration of ZO-1 was only observed when DON was used at 6.74 *μ*mol/L [6]. Claudin-3 was detected as a continuous lining around each untreated IPEC-J2 cell, in contrast to DON-contaminated cells in which reduced expression of tight junctional protein, claudin-3 was seen [5]. Several papers focused on the elucidation of the correlation between DON application and decrease in protein expression of claudin-3 and claudin-4 in intestinal epithelial cells [9–11]; however, only one finding suggests that DON affects claudin-1 expression at an elevated 20 *μ*mol/L concentration [12]. Changes in occludin localization after DON administration have not been completely elucidated yet.

T-2 could influence transcript levels of claudin-3, claudin-4, and occludin in human epithelial (Caco-2) cells when this mycotoxin was applied in micromolar concentrations [13]. Hence, the effects of T-2 on the assembly of TJ proteins have not been clarified yet.

The effects of DON on the modulation of cytokine production have been reported mostly based on the changes in mRNA levels. Kang et al. [8] observed that in proinflammatory cytokine, interleukin- (IL-) 6 gene expression was significantly increased in IPEC-J2 cells exposed to DON at 0.5–2 *μ*mol/L concentrations for 4 h. In accordance, Liu et al. [14] reported that IL-6 mRNA abundances of IPEC-J2 cells were elevated after 8 h incubation of cells with 4 *μ*mol/L DON. Guo et al. [15] also found that that 24 h treatment of IPEC-J2 cells with 1 *μ*mol/L DON led to the increase in IL-6 levels. However, there are controversial data regarding DON-induced mRNA abundances of IL-8 in IPEC-J2 cells. Liu et al. [14] found that DON at 4 *μ*mol/L concentration for 8 h could not trigger significant alterations in gene expressions of IL-8; however, it could cause downregulation of IL-8 levels at 0.25 *μ*mol/L. It was also ascertained that higher concentrations of DON (at 6.7 *μ*mol/L) did not change the expressions of IL-8 mRNA in IPEC-J2 cells [16]. In contrast, Cano et al. [17] proved that DON at 10 *μ*mol/L for 8 h significantly elevated the mRNA levels of IL-8. IL-8 production was also measured by Guo et al. [15], and it was reported that 1 *μ*mol/L DON significantly increased IL-8 contents in IPEC-J2 with 24 and 48 h incubation time.

It is widely accepted that the toxicity of trichothecene mycotoxins is dominantly caused by oxidative stress. Inside the cells, DON and T-2 activated the mitogen activated protein kinases (MAPK) [18], Janus kinase/signal transducers, activators of transcription (JAK/STAT) [19], and nuclear factor-kappa B (NF-*κ*B) [20] signaling pathways, which could lead to apoptosis. In addition to ribosomes, mitochondria are considered target to the trichothecene mycotoxin contaminations [21, 22]. Kang et al. [8] published that DON at 6.7 *μ*mol/L in IPEC-J2 cells could cause significant elevations in intracellular ROS levels after 24 h mycotoxin exposure.

The polyphenolic compounds have protective properties against oxidative stress-related disorders. The phenolic compounds can be subdivided into flavonoids and nonflavonoid substances [23], and rosmarinic acid (RA) belongs to the nonflavonoid group (Figure 1(c)). The efficacy of RA in the inhibition of lipid peroxidation was reported by Fadel et al. [24]. RA is also capable of decreasing some of the proinflammatory cytokine production *in vitro*. Based on an auditory cell line, HEI-OC1 experimental data RA inhibited IL-6 and IL-1*β* levels at 50 and 100 *μ*mol/L concentrations on cadmium- (Cd^2+^-) treated cells after 1 h of incubation [25]. Villalva et al. [26] found that RA-enriched extract to Caco-2 cells could reduce the secretions of IL-6, IL-1*β*, and tumor necrosis factor- (TNF-) *α*.

Humans and animals may be exposed simultaneously to different mycotoxins produced by *Fusarium* species. In our research, binary mixture of DON and T-2 was used (DT2) to assess toxicological effects of these fusariotoxins and to characterize putative preventive function of RA in the development of intestinal dysfunction *in vitro*.

The aims of our study were (i) to elucidate cytotoxicity of DON, T-2, DT2, and RA, (ii) to determine the impact of DT2 and RA on intestinal barrier integrity, and (iii) to assess IL-6- and IL-8-regulating and oxidative stress-inducing properties of mycotoxin combination and RA using IPEC-J2 cells. Moreover, an immunofluorescent study was also performed to monitor the changes in localization pattern of two TJ proteins, occludin and claudin-1, in IPEC-J2 cells exposed to DT2 in the absence and in the presence of RA.

## 2. Materials and Methods

### 2.1. Reagents

DON (molecular weight 296.319 g/mol), T-2 toxin (466.527 g/mol), and RA (360.31 g/mol) were purchased from Merck (Darmstadt, Germany). Dimethyl sulfoxide (DMSO) and acetonitrile were obtained from Thermo Fisher Scientific (Waltham, MA, USA). The final concentration of acetonitrile or DMSO in the cell culture medium was less than 0.5% (*v*/*v*).

### 2.2. Cell Culture

The porcine intestinal epithelial cell line, IPEC-J2 (ACC 701), was grown in 50% Dulbecco's Modified Eagle's Medium (DMEM) and 50% Ham's F12 Nutrient Mixture (Merck, Darmstadt, Germany) supplemented with 1.5 mmol/L HEPES, 5% fetal bovine serum (Biocenter, Budapest, Hungary), 1% insulin/transferrin/sodium selenite media supplement, 5 ng/mL epidermal growth factor, and 1% penicillin/streptomycin (all purchased from Invitrogen, Thermo Fisher Scientific, Waltham, MA, USA). The IPEC-J2 cell line was obtained from Dr. Jody Gookin, Department of Clinical Sciences, College of Veterinary Medicine, North Carolina State University, Raleigh, NC, USA. Cells were used between passages 40 and 42. Cells were maintained in 75 cm^2^ cell culture flasks with filter screw caps (Orange Scientific, Braine-l'Alleud, Belgium) at 37°C in a humidified atmosphere of 5% CO_2_. Complete culture medium was changed every other day.

### 2.3. Exposure of IPEC-J2 Cells to Mycotoxins and RA

IPEC-J2 cells were treated with DON or T-2 both apically and basolaterally. DON in a concentration range of 0–50 *μ*mol/L or T-2 in 0–50 nmol/L was used for 48 h and 72 h.

For assessing the impact of mycotoxin combinations, the following mixtures were prepared and added both apically and basolaterally: 1 *μ*mol/L DON + 5 nmol/L T − 2; 5 *μ*mol/L DON + 10 nmol/L T − 2; 10 *μ*mol/L DON + 5 nmol/L T − 2; and 10 *μ*mol/L DON + 10 nmol/L T − 2. Two incubation times were applied (48 h and 72 h).

IPEC-J2 cells, which obtained antioxidant treatments, were preincubated with RA for 24 h at 50, 100, 500, and 1000 *μ*mol/L. RA was dissolved in culture medium with 5% DMSO. Dissolved substances were filtered with syringe filters (Millex-GV, pore size: 0.2 *μ*m, Merck, Darmstadt, Germany) before application on the IPEC-J2 cells.

### 2.4. Evaluation of Cytotoxicity with Neutral Red Uptake Assay

Viability of IPEC-J2 cells was measured 48 h and 72 h after treatment with DON, T-2, DT2, and RA by neutral red (NR) uptake assay (Merck, Darmstadt, Germany). IPEC-J2 cells were incubated with mycotoxins and RA for 48 h and 72 h, respectively. The control cells were incubated only with phenol red-free DMEM. After the removal of the medium and washing, 45 mg/L NR solution was added [27] to the IPEC-J2 cells in plain phenol red-free medium for 2 h. After washing the IPEC-J2 cells, a destaining solution (ethanol/demineralized water/glacial acetic acid, 7.5/7.4/0.15, *v*/*v*/*v*) was applied for 10 min. The viability was measured at 540 nm using an ELISA Plate Reader (EZ Read Biochrom 400, Cambridge, UK).

### 2.5. Measurement of the Integrity of IPEC-J2 Cells

The measurement of TEER across epithelial monolayers is used to evaluate the integrity of the TJ barrier. IPEC-J2 cells were seeded on 6-well Transwell inserts (polyester, 0.4 *μ*m pore size, Corning, Merck, Darmstadt, Germany), and the seeding density was 1 x 10^6^ cells/well. Barrier function was evaluated after the cells reached confluent state and was measured with EVOM (Epithelial Tissue Volt/Ohmmeter) (World Precision Instruments, Berlin, Germany). The results were calculated as k*Ω* x cm^2^ by multiplying the values by the surface area of the monolayer (4.67 cm^2^).

### 2.6. Quantification of H_2_O_2_ Production

H_2_O_2_ production was monitored in IPEC-J2 cells by using the Amplex Red Hydrogen Peroxide Assay Kit (Invitrogen, Thermo Fisher Scientific, Waltham, MA, USA). In the presence of horseradish peroxidase, Amplex Red reagent reacts with H_2_O_2_ (in 1 : 1 stoichiometry) to produce a red fluorescent product called resorufin. After 48 h and 72 h, cell-free supernatants were taken from the basolateral compartment and were used for further experiments. 50 *μ*L of the collected cell-free supernatant was mixed with 50 *μ*L of the Amplex Red working solution according to the manufacturer's instructions. The fluorescence intensities were measured with a fluorometer (Victor X2 2030, Perkin Elmer, Waltham, MA, USA) using 560 nm excitation and 590 nm emission wavelengths.

### 2.7. Determination of Proinflammatory Cytokines Expression

IL-6 and IL-8 levels were determined in IPEC-J2 cell-free supernatants using porcine IL-6 (Aviva System Biology, San Diego, USA) and IL-8 sandwich ELISA kits (Merck, Darmstadt, Germany). To elucidate the cytokine levels after 48 h and 72 h treatment, the supernatants were treated according to the manufacturer's instructions and measured with an EZ Read Biochrom 400 microplate reader (Biochrom, Cambridge, UK) at 450 nm. Cytokine concentrations were calculated from the measured absorbance values and were expressed as means ± SDs.

### 2.8. Localization of Occludin and Claudin-1 Distribution *via* Immunofluorescent Staining

The localization of claudin-1 and occludin was assessed by confocal microscopy. Confluent IPEC-J2 cells were incubated with DT2 or DT2 + RA added apically and basolaterally for 72 h. Cells were fixed with 100% methanol (Met-OH, Merck, Darmstadt, Germany) and stained on the membrane inserts.

IPEC-J2 cells were blocked for 20 min at room temperature in bovine serum albumin solution (phosphate-buffered saline (PBS) buffer supplemented with 5% bovine serum albumin (BSA, Merck, Darmstadt, Germany)). Sections were incubated for 1 h at room temperature in the presence of anti-claudin-1 rabbit polyclonal primary antibody (1 : 200, Invitrogen, Thermo Fisher Scientific, Waltham, MA, USA) or anti-occludin rabbit polyclonal primary antibody (1 : 200, Merck, Darmstadt, Germany). The antibodies were previously diluted in 5% BSA solutions. Then the inserts were incubated with Alexa Fluor 546 conjugated anti-rabbit IgG secondary antibodies (1 : 200, Invitrogen, Thermo Fisher Scientific, Waltham, MA, USA), which were diluted in PBS. The sialic acid residues in IPEC-J2 cell membrane were stained with wheat germ agglutinin conjugated with Alexa Fluor 488 (1 : 200 diluted in PBS, WGA Alexa Fluor 488, Invitrogen, Thermo Fisher Scientific, Waltham, MA, USA) for 10 min, and cell nuclei were stained in blue using 4',6-diamidino-2-phenylindole (DAPI) (1 : 500 diluted in PBS, Invitrogen, Thermo Fisher Scientific, Waltham, MA, USA) for an additional 10 min.

Between incubations, the inserts were washed in PBS for 3 x 5 min. Inserts were attached on glass slides using fluorescent mounting medium (Dako, Agilent Technologies, Glostrup, Denmark). The claudin-1 samples were analyzed using a Zeiss confocal microscope 63x Plan Apochromat 63x/1.4 Oil DIC M27 (Zeiss LSM 710 Confocal Microscope, Oberkochen, Germany) while the occludin localization was detected with Leica confocal microscope; lenses were 63x/1.4 Oil (Leica SP2 Confocal Microscope, Wetzlar, Germany).

### 2.9. Statistical Analysis

The statistical analysis of the results was performed by using the R Core Team (2016) (R: A language and environment for statistical computing (R Foundation for Statistical Computing, Vienna, Austria)). Differences between groups were analyzed by one-way ANOVA coupled with the post hoc Tukey test for multiple comparisons, where data were of normal distribution and homogeneity of variances was confirmed. ∗*p* < 0.05; ∗∗*p* < 0.01, and ∗∗∗*p* < 0.001 were considered statistically significant.

## 3. Results

### 3.1. Cytotoxicity of DON, T-2, DT2, and RA

**Figure 2:**
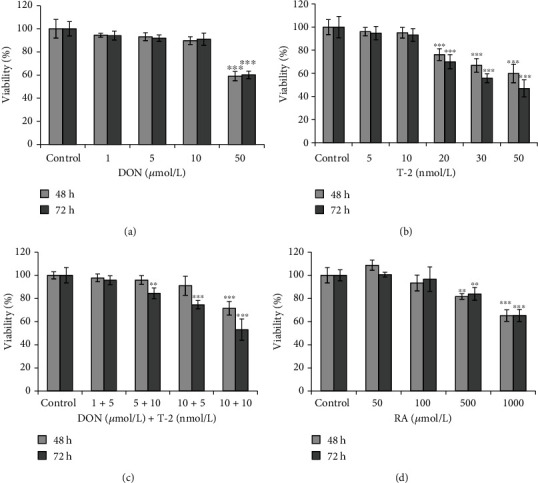
Effects of (a) DON, (b) T-2, (c) DT2, and (d) RA on viability % of the differentiated IPEC-J2 cells measured by NR assay. Incubation times were 48 h and 72 h. Significant differences were found between untreated samples and cells exposed to 50 *μ*mol/L DON or 20 nmol/L T-2. In the case of DT2 treatment, addition of 1 *μ*mol/L DON and 5 nmol/L T-2 to IPEC-J2 cells did not influence cell viability significantly for 48 h and 72 h. RA appeared to be cytotoxic at higher concentrations (at 500 and at 1000 *μ*mol/L). Data are presented as viability%means ± SDs (*n* = 8, ∗∗*p* < 0.01 and ∗∗∗*p* < 0.001).

The cytotoxic effects of DON, T-2, DT2, and RA on IPEC-J2 cells were evaluated over 48 h and 72 h (Figure 2). DON and T-2 were applied in a concentration range of 0–50 *μ*mol/L and 0–50 nmol/L, respectively. Significant cell death was observed upon exposure of cells to DON at 50 *μ*mol/L after 48 h and 72 h incubation (*p* < 0.001) (Figure 2(a)). T-2 showed cytotoxic effects at 20 nmol/L and at higher concentrations after 48 h and 72 h treatments (*p* < 0.001) (Figure 2(b)). Mycotoxin combination (DT2) was also tested (Figure 2(c)). It was found that when IPEC-J2 cells were treated with DON and T-2 simultaneously, the 5 *μ*mol/L DON + 10 nmol/L T − 2, 10 *μ*mol/L DON + 5 nmol/L T − 2, and 10 *μ*mol/L DON + 10 nmol/L T − 2 treatments were toxic to the cells significantly after 72 h incubation (*p* < 0.01 and *p* < 0.001). The effect of RA on cell viability was tested in concentration range of 50–1000 *μ*mol/L (Figure 2(d)). Treatment with RA at 500 and 1000 *μ*mol/L significantly decreased cell viability after 48 h and 72 h exposure (*p* < 0.01 and *p* < 0.001); however, RA did not deteriorate cell viability at lower concentrations (50 and 100 *μ*mol/L). For further investigations, we applied the noncytotoxic 1 *μ*mol/L DON + 5 nmol/L T − 2 mycotoxin combination and RA at 50 *μ*mol/L.

### 3.2. Cell Membrane Integrity Changes after Mycotoxin and RA Exposure

**Figure 3:**
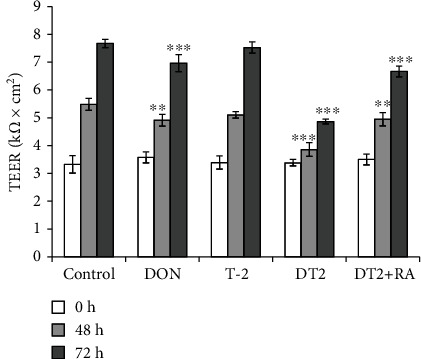
Transepithelial electrical resistance (TEER) measurements of IPEC-J2 monolayers. Cells were incubated with 1 *μ*mol/L DON, with 5 nmol/L T-2, with DT2 (1 *μ*mol/L DON + 5 nmol/L T − 2), and with DT2 + RA (1 *μ*mol/L DON + 5 nmol/L T − 2 + 50 *μ*mol/L RA) (24 h preincubation) for 48 h and 72 h. TEER values are expressed in k*Ω* × cm^2^. ∗∗*p* < 0.01 and ∗∗∗*p* < 0.001 compared to the control values. Data are presented as TEER means ± SDs (*n* = 9).

To determine the barrier disrupting effects of DON, T-2, and DT2 and the putative barrier-reinforcing effect of RA, TEER measurements were carried out using 48 h and 72 h treatment times (Figure 3). Incubation of cells with 5 nmol/L T-2 did not alter TEERs of cell monolayers, but 48 h DON and DT2 administration caused significant decrease in TEER values (*p* = 0.003 and *p* < 0.001). TEERs of IPEC-J2 cells treated with DT2 + 50 *μ*mol/L RA showed lower TEERs compared to those of controls (*p* = 0.006), but they were significantly elevated to decreased TEERs of DT2-treated samples (*p* < 0.001) after 48 h exposure. The detrimental effect of DT2 could be suppressed partially with the application of RA after 48 h and 72 h.

### 3.3. Changes in Extracellular H_2_O_2_ Production after Mycotoxin and RA Treatments

**Figure 4:**
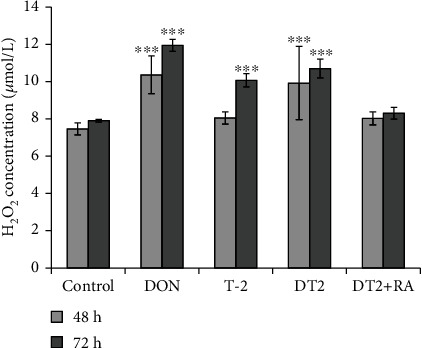
The changes of H_2_O_2_ production after incubation of IPEC-J2 cells with 1 *μ*mol/L DON, 5 nmol/L T-2, DT2 (1 *μ*mol/L DON + 5 nmol/L T − 2), and DT2 + RA (1 *μ*mol/L DON + 5 nmol/L T − 2 + 50 *μ*mol/L RA) (24 h preincubation) for indicated time periods (48 h and 72 h). ∗∗∗*p* < 0.001 compared to the control values. Data are presented as means ± SD (*n* = 8).

The effect of the DON, T-2, DT2, and RA treatments on the extracellular H_2_O_2_ production of the IPEC-J2 cells was determined. The cell-free supernatants were collected after 48 h and 72 h (Figure 4). Based on the results, after 48 h incubation of cells with 1 *μ*mol/L DON and DT2, the extracellular H_2_O_2_ levels were significantly increased (in each case *p* < 0.001). When DT2-treated samples were compared with those exposed to DT2 + RA, significant differences were measured (*p* < 0.001). The H_2_O_2_ contents produced by DT2 + RA-treated IPEC-J2 cells did not differ from the H_2_O_2_ levels from control cells. DON, T-2, and DT2 induced significant increase in extracellular H_2_O_2_ levels after 72 h treatment. DT2-caused oxidative stress could be quenched effectively with the pretreatment of cells with RA at 50 *μ*mol/L for 24 h (*p* < 0.001).

### 3.4. The Effect of DT2 and RA on IL-6 and IL-8 Levels

**Figure 5:**
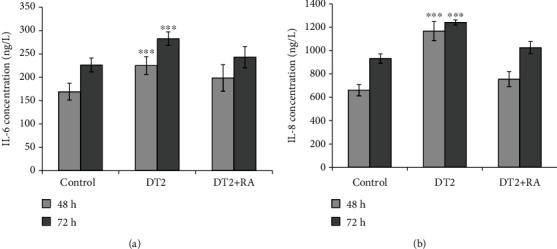
Measured changes of (a) IL-6 and (b) IL-8 of IPEC-J2 cells. The cytokine concentrations (ng/L) in the cell-free supernatants were calculated using porcine sandwich ELISA kits. Cells were treated with DT2 (1 *μ*mol/L DON + 5 nmol/L T − 2) and DT2 + RA (1 *μ*mol/L DON + 5 nmol/L T − 2 + 50 *μ*mol/L RA) (24 h preincubation) for 48 h and 72 h. ∗∗∗*p* < 0.001 compared to the control values. Data are presented as means ± SD (*n* = 10).

The IL-6 levels (Figure 5(a)) were elevated after DT2 (*p* < 0.001) exposure, and overproduction was completely inhibited by DT2 + RA treatments for 48 h and 72 h. There were no significant differences in IL-6 levels between control samples and the DT2 + RA-treated cells (*p* = 0.145, 48 h, and *p* = 0.711, 72 h). The IL-8 levels (Figure 5(b)) were also increased significantly in DT2-treated IPEC-J2 cells using 48 h and 72 h incubation times (both *p* < 0.001). DT2 + RA at 50 *μ*mol/L could stabilize perturbed IL-8 levels triggered by application of DT2. There were no significant differences in IL-8 levels between RA-protected DT2 samples and control-treated cells (*p* = 0.256, 48 h, and *p* = 0.368, 72 h).

### 3.5. Cellular Distribution of Claudin-1 and Occludin in IPEC-J2 Cells Exposed to DT2 and RA

**Figure 6:**
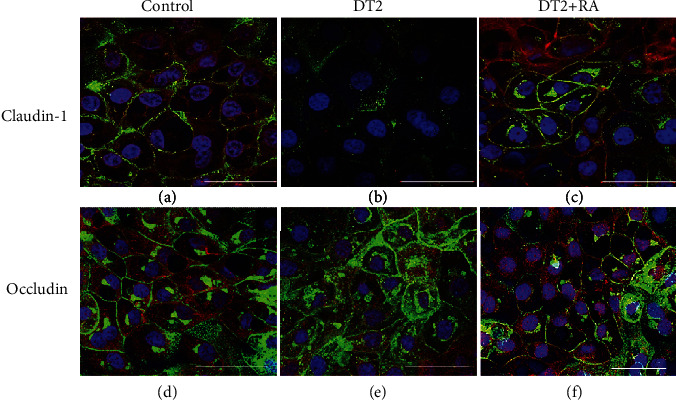
Effects of DT2 and DT2 + RA (RA pretreatment for 24 h) on the localization pattern of (a–c) claudin-1 and (d–f) occludin using immunofluorescent staining. Differentiated IPEC-J2 cells were cultured on membrane inserts for 10 days then were exposed to DT2 (1 *μ*mol/L DON + 5 nmol/L T − 2) or to DT2 + RA (1 *μ*mol/L DON + 5 nmol/L T − 2 + 50 *μ*mol/L RA); both treatments were applied apically and basolaterally for 72 h. Cells were stained for occludin and claudin-1 (Alexa Fluor 546, red). Cell nuclei were stained with DAPI (blue), and cell membranes were labelled with wheat germ agglutinin (Alexa Fluor 488, green). In controls and in DT2 + RA-treated samples, claudin-1 and occludin were colocalized with wheat germ agglutinin. White scale bar shows 50 *μ*m.

Localization of claudin-1 (Figures 6(a)–6(c)) and occludin (Figures 6(d)–6(f)) in TJ assembly was assessed in untreated control and in DT2-treated IPEC-J2 cells using immunofluorescence staining. The cells were investigated 72 h after DT2 and DT2 +RA treatments. The localization pattern of claudin-1 significantly changed when DT2 was continuously administered. The loss of membranous claudin-1 proteins from TJ was observed in the form of discontinuous membrane pattern in contrast to the distribution of those in control cells (Figures 6(a) and 6(b)). This phenomenon might explain TEER changes observed when DT2 was administered to the IPEC-J2 cells for 72 h.

In controls, occludin localized in membranes of polarized IPEC-J2 cells (Figure 6(d)) and when cells were exposed to DT2 (1 *μ*mol/L DON + 5 nmol/L T − 2), occludin maintained cell membranous presence (Figure 6(e)). RA given simultaneously with DT2 seemed to preserve the integrity of the TJ protein assembly by maintaining the belt-like structures of occludin and claudin-1 (Figures 6(c) and 6(f)). DT2 + RA treatment (1 *μ*mol/L DON + 5 nmol/L T − 2 + 50 *μ*mol/L RA) resulted in similar continuous lining of occludin and claudin-1 around each cell, similarly to that detected in untreated cells.

## 4. Discussion

Due to the natural cooccurrence of fusariotoxins in food and in feedstuffs, the toxicological evaluation of the impact of combined mycotoxins on gut barrier appeared to be of key importance. Most of the studies focus on separately added mycotoxin; however, *in vitro* interaction exists between fusariotoxins in terms of cell viability [28].

In our study, the DON and T-2 binary combination was firstly used on the porcine nontumorigenic nontransformed jejunal epithelial cells to elucidate the impact of these two trichothecene mycotoxins on barrier integrity of IPEC-J2 cell monolayers cultured on permeable support membranes. Until now only a few *in vitro* models have been used to perform risk assessments in the case of cooccurrence of DON and T-2 mycotoxins. Lei et al. [29] found that treatment with DON (2.696 *μ*mol/L) and T-2 (21.4 nmol/L) increased oxidative stress thus inducing apoptosis in culture models, in chondrocytes and hepatic/tubular epithelial cell lines. Ruiz et al. [30, 31] examined the impact of DON and T-2 mycotoxin combination on immortalized hamster ovarian cells (CHO-K1) and on mammalian kidney epithelial (Vero) cell lines, and it was found that they acted antagonistically at 24 h, 48 h, and 72 h of exposure. In contrast, Ficheux et al. [32] concluded that the toxicity of combination was greater than the individual toxicity of each mycotoxin in the case of DON and T-2 applied for 14 days on white blood cells progenitor cells, colony-forming unit of granulocyte/monocyte (CFU-GM). Additive effect of DON and T-2 on inhibition of platelet aggregation was reported possibly via similar biochemical mechanisms, when porcine platelet suspensions were exposed to this mycotoxin combination [33].

Cell viability assays can be used for better *in vitro* toxicological evaluation of the harmful effects of DON and T-2 and their combinations. Goossens et al. [7] determined the ratio of viable, apoptotic, and necrotic cells after incubation of IPEC-J2 cells with different concentrations of DON and T-2 for 72 h using flow cytometric technique. IC_50_ values for DON were 23.5 *μ*mol/L and 20.4 nmol/L for T-2 [7], respectively; therefore, decrease in TEER might be correlated with the cytotoxic effects of mycotoxins. Springler et al. [12] performed NR assay and lactate dehydrogenase (LDH) test for establishing the effect of DON on the viability of differentiated IPEC-J2 cells. It was found that DON could reduce significantly cell viability at 50 *μ*mol/L after 24 h exposure using NR assays, and LDH test could detect 33% change in cell viability after 48 h incubation of IPEC-J2 cells with DON. Vandenbroucke et al. [34] reported that undifferentiated and differentiated IPEC-J2 cells have different sensitivities to DON contamination. In case of nonpolarized IPEC-J2, cell death was significant upon 24 h addition of DON at as low as 0.8425–33.7 *μ*mol/L concentrations. In contrast, differentiated IPEC-J2 maintained viability in this concentration range; however, DON could induce significant reduction in TEER of cell monolayers. T-2 toxicity was evaluated by Verbrugghe et al. [35], and it was confirmed that IC_50_ values of T-2 mycotoxin for undifferentiated and differentiated IPEC-J2 cells were 12.198 nmol/L and 395.9 nmol/L, respectively. In our work, we found firstly that DT2 combination did not affect IPEC-J2 cell viability at 1 *μ*mol/L DON + 5 nmol/L T − 2 concentration after 72 h exposure.

It was also reported that the addition of DON at 3.37 *μ*mol/L and T-2 at 21.4 nmol/L concentration for 72 h decreased TEER in a time- and concentration-dependent manner, and when the cells were incubated with cytotoxic concentrations of these mycotoxins, increased passage of doxycycline and paromomycin was measured across the IPEC-J2 cell monolayer [7]. Basolateral application of DON at 6.74 *μ*mol/L caused significant decrease in TEER of IPEC-J2 cell monolayers after 24 h exposure [5]. In accordance with this, Springler et al. [12] confirmed that DON reduced TEER significantly at 5–20 *μ*mol/L after 24 h incubation. It is widely accepted that T-2 contamination can cause pathological gut lesions, but only few scientific data available for assessing the underlying mechanisms in the background of nanomolar T-2-related barrier dysfunction. The presence of T-2 contamination triggered transepithelial passage of *Salmonella typhimurium* across IPEC-J2 cell monolayers, which was proven by application of T-2 in lower concentration range of 1.6–10.5 nmol/L without detecting changes in TEER values after 24 h incubation [36]. In this work, we firstly reported that DT2 induced a significant decrease in TEERs of IPEC-J2 cell monolayers exposed to binary mixture of DT2 for 48 h and 72 h, which could be compensated partially by application of RA at 50 *μ*mol/L. Vergauwen et al. [37] concluded that preincubation of cells with 200, 400, and 600 *μ*mol/L RA for 18 h could reinforce the IPEC-J2 cell monolayer integrity after peroxide challenge. This is in good agreement with our data as RA could also play a key role in strengthening the IPEC-J2 monolayer barrier integrity after exposure to binary mixture of fusariotoxins DT2 in our study.

The overproduction of cytokines plays a key defensive role in innate immune responses against noxious stimuli in epithelial cells [38, 39]. Wan et al. [40] reported that DON caused elevations in relative abundances of IL-6 and IL-8 in IPEC-J2 cells exposed to 0.5–2 *μ*mol/L DON for 48 h. The impact of T-2 on cytokine levels in intestinal epithelial cells has not been widely studied yet. T-2 applied in concentration range of 4.29–275 nmol/L appeared to upregulate IL-8 levels in Caco-2 cells after 20 h exposure [41]. In accordance with these results, we found that IL-6 and IL-8 overproduction occurred when IPEC-J2 cells were incubated with DT2 combination for 48 h and 72 h. In our study, these elevations in IL-6 and IL-8 levels were prevented with pretreatment of cells with 50 *μ*mol/L concentration of RA for 24 h.

The mode of action of DON and T-2 involves the induction of oxidative stress, which might suggest the beneficial role of plant-derived polyphenolic compounds such as RA in the prevention of fusariotoxin-induced intestinal damage. Recent *in vivo* studies have shown that dietary flavonoid supplementation in pig feed could reduce oxidative stress and inflammation, and thus, it could improve the overall performances of the pigs [42–44]. Zha et al. [43] proved that administration of flavone-type baicalin with copper could maintain optimal growth and increase antioxidant capacity in piglets fed with DON-contaminated feeds. Baicalin zinc supplementation could also restore DON-triggered impairment in nutrient absorption and provide antioxidant defense against excessive oxidative stress [44]. Antioxidant properties of RA with similar structural polyphenolic backbone were previously proven in *in vitro* and *in situ* experiments. Vergauwen et al. [37] carried out experiments with IPEC-J2 cells using various concentrations of RA, and it was confirmed that RA at higher concentrations (200, 400, and 600 *μ*mol/L) could reduce intracellular ROS. In our study, we proved that extracellular H_2_O_2_ levels were successfully quenched by preincubation of RA at 50 *μ*mol/L in IPEC-J2 cells treated with DT2.

There have been several studies involving the impact of fusariotoxins on localization or expression of TJ proteins. Untreated IPEC-J2 cells showed homogenous, intense membranous occludin and claudin-1 positivity. Springler et al. [12] reported that DON did not affect occludin and claudin-4 levels at 20 *μ*mol/L for 72 h, but it significantly reduced claudin-1 and claudin-3. This observation is in contrast to that of Gu et al. [45], since they reported that lower concentration of DON (6.74 *μ*mol/L) for 48 h could decrease occludin expression. However, until now, there have not been any documented data considering the impact of DT2 on the localization pattern of TJ proteins such as occludin and claudin-1 in IPEC-J2 cells. Based on our immunofluorescent findings, DT2 applied for 72 h did not alter occludin localization pattern; however, claudin-1 was lost from cell membrane observed as disruption in continuous lining of claudin-1 in cell membrane. Occludin appears to be one of the key constituents of TJ assembly; however, the formation of TJ does not only depend on occludin itself as it was proven by Suzuki [46]. Thereby, it can be assumed that changes in TEER induced by DT2 treatment can at least partially be correlated with relative claudin-1 absence from TJ strands. Qiang [47] reported that RA at 50 *μ*mol/L upregulated the mRNA expressions of ZO-1, ZO-2, claudin-1, and occludin in Caco-2 cells. It is in good agreement with our findings that RA could promote the membranous presence of claudin-1 in IPEC-J2 cells which were exposed to DT2.

## 5. Conclusions

In conclusion, this study demonstrated that binary mixture of DT2 at noncytotoxic concentrations could deteriorate barrier integrity of IPEC-J2 cells, and it could elevate the levels of inflammatory IL-6 and IL-8. These harmful effects could be alleviated by 24 h preadministration of polyphenolic RA. It was also shown that DT2-promoted oxidative stress could be effectively quenched by RA application. Moreover, changes in protein TJ assembly including claudin-1 redistribution were detected in DT2-treated cells which could be restored by RA addition. Therefore, RA appeared to have anti-inflammatory, antioxidant, and barrier-reinforcing potential in the prevention of DT2-caused detrimental intestinal effects *in vitro*.

## Data Availability

The data used to support the findings of this study are available from the corresponding author upon request.
